# Selective Laser Sintering (SLS), a New Chapter in the Production of Solid Oral Forms (SOFs) by 3D Printing

**DOI:** 10.3390/pharmaceutics13081212

**Published:** 2021-08-06

**Authors:** Yanis A. Gueche, Noelia M. Sanchez-Ballester, Sylvain Cailleaux, Bernard Bataille, Ian Soulairol

**Affiliations:** 1 ICGM, University Montpellier, CNRS, ENSCM, 34000 Montpellier, France; yanis-abdelhamid.gueche@etu.umontpellier.fr (Y.A.G.); noelia.sanchez-ballester@umontpellier.fr (N.M.S.-B.); sylvain.cailleaux@gmail.com (S.C.); bernard.bataille@umontpellier.fr (B.B.); 2 Department of Pharmacy, Nîmes University Hospital, 30900 Nimes, France

**Keywords:** selective laser sintering, 3D printing, solid oral forms, personalized medicine, printability, orally disintegrating forms

## Abstract

3D printing is a new emerging technology in the pharmaceutical manufacturing landscape. Its potential advantages for personalized medicine have been widely explored and commented on in the literature over recent years. More recently, the selective laser sintering (SLS) technique has been investigated for oral drug-delivery applications. Thus, this article reviews the work that has been conducted on SLS 3D printing for the preparation of solid oral forms (SOFs) from 2017 to 2020 and discusses the opportunities and challenges for this state-of-the-art technology in precision medicine. Overall, the 14 research articles reviewed report the use of SLS printers equipped with a blue diode laser (445–450 nm). The review highlights that the printability of pharmaceutical materials, although an important aspect for understanding the sintering process has only been properly explored in one article. The modulation of the porosity of printed materials appears to be the most interesting outcome of this technology for pharmaceutical applications. Generally, SLS shows great potential to improve compliance within fragile populations. The inclusion of “Quality by Design” tools in studies could facilitate the deployment of SLS in clinical practice, particularly where Good Manufacturing Practices (GMPs) for 3D-printing processes do not currently exist. Nevertheless, drug stability and powder recycling remain particularly challenging in SLS. These hurdles could be overcome by collaboration between pharmaceutical industries and compounding pharmacies.

## 1. Introduction

Presently, medicines are mainly delivered to patients in the form of pharmaceutical specialties manufactured by large pharmaceutical companies [1]. However, this “one-size-fits-all” approach, promoted by the industrial revolution, is restrictive because not all individuals react in the same way to a disease and hence to a pharmacological treatment [2]. In fact, intrinsic factors such as age [3,4], sex [5,6], and genetics [7], as well as extrinsic factors such as the environment [8], are involved in both the clinical manifestation of a disease and the pharmacological response. This is where “personalized medicine” comes in. This novel paradigm is defined according to the Precision Medicine Initiative as “an emerging approach for disease treatment and prevention that takes into account individual variability in genes, environment and lifestyle for each person” [9]. Precision medicine implies the administration of the appropriate drug at the right dosage with an adapted release mode. Hence, it requires the formulation of this precise drug dosage into a pharmaceutical form [10]. Nevertheless, when it comes to manufacturing a variety of specific dosages on a small scale, industrial machines that are dedicated to mass production may not be the best contenders. Fortunately, not all innovative technologies are aimed solely at improving productivity; some also focus on creating products that meet the specific needs of each individual [11], such as 3D printing.

This state-of-the-art technology allows the production of objects of different sizes and shapes, according to a pre-established design. Therefore, it offers greater flexibility than conventional processes [12,13,14]. Although 3D printing is a well-established reality in the fields of architecture, aeronautics and recently medical devices, in the drug market there is only one pharmaceutical specialty produced by 3D printing and approved by the FDA (2015): Spritam^®^ (Levetiracetam) [15]. Three-dimensional printing could help improve individualized oral therapy, especially solid oral forms (SOFs), which currently present limited options for individually designed doses. Indeed, while liquid forms can be easily dosed using oral syringes, they also present main disadvantages such as poor stability, administration errors and unpleasant taste. In contrast, solid forms, especially tablets, present higher stability, but the possibilities of dosing them individually are limited [16]. 

Different 3D printing techniques have been investigated to produce SOFs such as binder and material jetting [17,18,19], fused deposition modeling (FDM) [20,21,22], semi-solid extrusion (SSE) [23,24], stereolithography (SLA) [25,26] and selective laser sintering (SLS) [27]. Although since 2014 the majority of the articles has focused on FDM technology, describing it as the most promising [28]; SLS has gained considerable attention in the last four years (from 2017 to 2020) accumulating a total of 14 research articles [27,29,30,31,32,33,34,35,36,37,38,39,40,41] and two reviews [42,43].

SLS is classified under the Powder Bed Fusion category according to the ASTM (American Society for Testing Material) [44]. It involves the building of objects by necking powder particles using the energy provided by a laser [45]. This additive manufacturing technique presents many benefits such as high resolution, the possibility of recycling the powder and the absence of pre-processing [43]. Moreover, pharmaceutical manufacturing requires a higher threshold of quality and safety. This justifies the relevance of using pharmaceutical grade powders, which are recognized to be safe for the human body, but these materials should also be printable and remain stable during the printing process. The literature on SLS provides knowledge of the requirements for printability and stability of the feedstock materials [46,47,48]. However, switching from conventional powders to pharmaceutical powders can be challenging [49,50,51].

The aim of this article is to review the research conducted on SLS 3D printing of pharmaceutical SOFs from 2017 to 2020. Hence, the printers and materials employed, the printing parameters explored and the main benefits of using SLS for the preparation of SOFs are presented here. Although previous reviews have focused on the presentation of already demonstrated pharmaceutical applications, this article discusses their limitations and suggests potential areas of research to improve them. Furthermore, technical and regulatory challenges are highlighted, with a particular focus on printability. Finally, the work concludes by proposing a model for the establishment of SLS in the pharmaceutical landscape.

## 2. Overview of Studies

Fourteen publications on SLS applied to the pharmaceutical research have been identified on the MEDLINE^®^ database from 2017 to 2020. The objectives, used materials, dimensions of the printlets and drug release profiles for each study are presented in Table 1. The majority of the studies [27,29,30,31,32,34,35,36,40] were issued by a team based in UCL School of Pharmacy (London) in partnership with the startup FabRx [52]. They are the developers of the trademark “Printlets^®^” which is a contraction of the words “print” and “tablets” to qualify SOFs produced by 3D printing. In this review, as authors we decided to use the word “printlets” to refer to printed SOFs rather than tablets. In our opinion, “tablets” should be reserved to SOFs manufactured by compression and a consensus will need to be found on the denomination of SOFs produced by 3D printing techniques by the competent authorities.

It is interesting to note that some research work on the development of drug-delivery devices (DDDs) by SLS attempting to modify their functional properties was already conducted before the production of SOFs. Although the aim of this paper is not to review the work conducted on the production of DDDs, it is important to comment on the studies of SLS on SOFs in the light of the previous DDD choice of biopolymers and drugs. These DDDs were produced using biodegradable and biocompatible polymers including polycaprolactone (PCL) [53,54,55,56,57,58,59], polylactic acid (PLA) [53,60], polyethylene (PE) [61,62,63], polystyrene (PS) [64] and cellulose derivatives [65].

## 3. Materials and Equipment

### 3.1. SLS PrInt.er and Process Parameters

**Figure 1:**
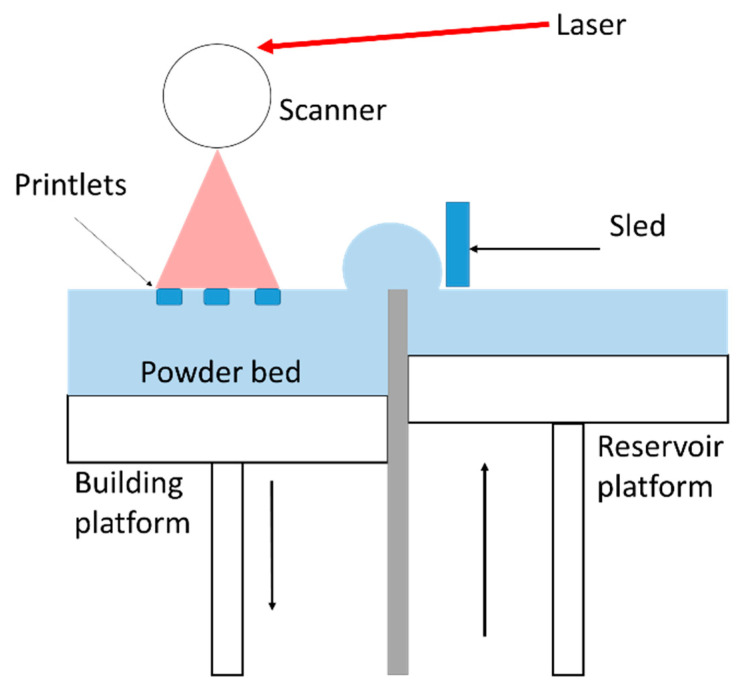
Schema of the SLS printer.

It is interesting to highlight that all the 14 reviewed articles were conducted with SLS printers equipped with a blue diode laser (445–450 nm), and 13 of them [27,29,30,31,32,33,34,35,36,37,38,39,40] report the use of the brand Sintratec kit (AG, Brugg, Switzerland). Templates of printlets were all designed using a CAD software, then saved as STL files before transferring them to the 3D printer software. Figure 1 illustrates the sintering process. First, powder is fed in the reservoir platform then distributed by a sled all over the building area to create a flat homogenous layer. Prior sintering, the printer needs to be heated to warm the powder. Printing per se starts by the activation of the blue diode laser which scans the powder bed in a specific pattern along the X and Y axes according to the pre-established design of the object. Powder particles are fused partially or completely together depending on the amount of transmitted energy. Next, the printing bed is lowered, and another layer of powder is deposited over the previously sintered layer allowing the building of the object along the Z axis. These steps are repeated until finalization of the object. At the end, the printed dosage forms are removed from the build platform then brushed off from their excess of powder. The powder that has not been consolidated remains in place and serves as support to the object during its building, which is very advantageous compared to other 3D printing techniques where supplementary support structures need to be built [48].

The blue diode laser (λ = 445 nm) of the Sintratec kit has a lower power (2.3 W) compared to other conventional lasers used in SLS such as CO_2_ and Nd-YAG lasers, whose power can reach 500 W [66]. This represents a primary advantage for the production of SOFs; since the input energy is relatively low, the active ingredients are less susceptible to undergo thermal degradation.

In SLS, the degree of sintering of an object is governed by multiple parameters, but mainly directed by the energy density (ED), which is the amount of energy transmitted per volume unit [67]. This critical parameter depends on four process parameters as shown by the empirical equation [68]:

$$ED\;\left(J/mm^{3}\right)=\frac{LP}{\left(SS\;\times \;HS\times LT\right)}$$

where *LP*, *SS*, *HS* and *LT* are respectively: laser power, scanning speed, hatch spacing and layer thickness.

The laser power in the Sintratec kit is not tunable. Hence, an alternative way to modulate the amount of energy transmitted to the powder is by varying the scanning speed or hatch spacing. The slower is the scanning, the longer is the interaction between the laser and the particles leading to a higher energy input [48]. Moreover, the hatch space corresponds to the distance between two consecutive sintered lines at the same layer. Therefore, a short hatch space will result in a better energy transfer [38,43].

Furthermore, it has also been shown that both layer thickness and process temperature affect the coalescence behavior between powder particles [48]. It is worth noting that while the influence of process temperature has been assessed [33], the effect of layer thickness on the properties of dosage forms has not been explored.

### 3.2. Raw Materials

SLS feedstock is based on powder, which is common to conventional pharmaceutical production methods such as tableting and granulation. The pre-printing step, often necessary for other 3D printing techniques such as FDM, is not required in SLS. In this process, the different components of the formulation are previously mixed and then the mixture is loaded directly into the printer. Thus, while FDM requires the formulation of filaments with excipients and active ingredients usually through the use of a thermal process known as hot-melt extrusion (HME) [69], SLS is a single step process.

#### 3.2.1. Polymers

Like FDM, SLS requires the use of thermoplastic polymers as matrices to carry drugs. This type of polymers present the ability to be processed and remolded upon thermal variations (heating and cooling) [70]. Before sintering, the heating temperature is set below the melting temperature for semi-crystalline polymers or below the glass transition temperature for amorphous polymers [48]. Then, the laser, depending on its scanning speed, will act as a final push to more or less fuse the powder particles. Several pharmaceutical thermoplastic polymers widely used for HME and FDM have also been explored for SLS such as copovidone (Kollidon VA64) [29,33,35,36,37,38], PEG-PVA (Kollicoat IR) [27,32,39,40], hydroxypropyl methylcellulose (HPMC) [29,31,41], ethylcellulose [30,32,41], acrylic polymers (Eudragit) [27,30,31,41] and polyethylene oxide (PEO) [30,34]. The frequent use of copovidone in SLS is due to two of its specific characteristics [71]. On one hand, it exhibits a low glass transition temperature which promotes the reduction of processing temperatures. On the other hand, it provides an erodible instant release matrix, which allows the production of fast disintegrating dosage forms. 

**Figure 2:**
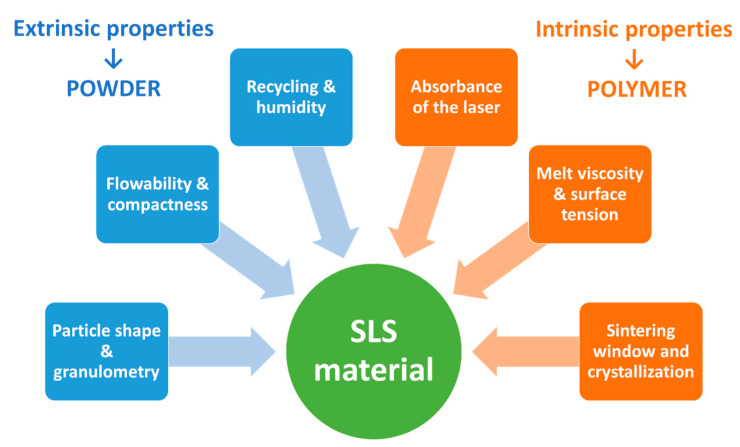
Main critical properties for the printability of polymeric powders in SLS.

However, not all polymer powders may be suitable for SLS, as they must meet a combination of intrinsic and extrinsic properties required for the SLS 3D printing process [72] (Figure 2). Intrinsic properties, such as melt viscosity, are related to the chemical structure of the polymer, while extrinsic properties, such as flowability, refer to the physical characteristics of the powder [73].

##### Intrinsic Properties

Absorbance at the Laser Wavelength

Since the thermal energy source is a laser beam, the particles need to absorb light at the same wavelength as the printer laser in order to sinter and form junctions [74]. However, in the case of the blue diode laser, none of the tested polymers absorb at the wavelength of 445 nm thus requiring the systematic incorporation of an absorbance enhancer such as Candurin^®^ to favor the sintering process [27,29,30,31,32,33,34,35,36,37,38,39,40]. Two types of the metallic colorant Candurin^®^ have been used, Gold Sheen [27,29,30,31,32,34,35,36,38,40] and NXT ruby red [33,39]. Concerning to the ratio used, all works confirmed that 3% of Candurin^®^ guarantees optimal sintering. Studies conducted on the evaluation of absorbance of different material at variable SLS laser wavelengths have shown that the absorbance of metals (titanium, aluminium) improves at low wavelengths (UV-visible domain) and that the absorbance of polymers tends to increase at high wavelengths (infrared domain) [74]. This explains the aptitude of the metallic dye to compensate for the lack of absorbance of polymers at 445 nm. More recently, other colorants were assessed and Tartrazine lake at 0.2% has been proved to be efficient in ensuring the sintering of pharmaceutical polymers with a blue diode laser [41].

Furthermore, Candurin^®^ is a safe dye used in the pharmaceutical industry as a coating agent for tablets [75]. Its components (titanium dioxide, potassium aluminium silicate and iron oxide), although recognized as safe by the FDA when taken at low doses, their safety remains still controversial [76,77]. Switching from a laser operating in the blue region of the spectrum to one working in the IR region of the spectrum may obviate the need for an absorbance enhancer, as many biocompatible and biodegradable polymers has been found to be sinterable with a CO_2_ laser [55,61,78] due to the presence of C-H bonds which absorb at the wavelength of the laser (λ = 10.6 µm) [73]. Thus, health risks among users can be minimized and possible physical-chemical interactions between the components of the formulation can be prevented. Moreover, absorbance is not only related to the chemical structure of the polymer, but also depends on the properties of the powder, e.g.,: a loose powder absorbs more than a dense one [79].

Solid State

For polymeric powders, the main mechanism of particle consolidation is liquid phase sintering, resulting from the partial or total melting of the powder particles. The molten polymer, driven by capillarity action, spreads within the powder and enables the formation of junctions between adjacent powder particles [80]. Therefore, low melt viscosity and high melt surface tension are desirable to achieve successful sintering. Those thermal and rheological properties are very different depending on whether the polymer is semi-crystalline or amorphous [73].

Semi-crystalline polymers melt when subjected to laser energy resulting in a large decrease in viscosity and an increase in density. This explains the better mechanical properties exhibited by sintered parts obtained using semi-crystalline polymers. Furthermore, in the case of semi-crystalline polymers, the temperature of the chamber is generally kept constant, below their melting point but above their crystallization point to reduce potential stresses and deformations caused by heterogeneous crystallization and rapid cooling. Hence, crystallization of the consolidated printed layers will only occur when the printing process is completed. On the other hand, amorphous polymers, which do not have melting or crystallization point, undergo a less precise phase transition over a broader temperature range above their glass transition point, allowing them to soften gradually. This results in reduced stresses and distortions that are critical for part accuracy, but also in a higher melt viscosity compared to semi-crystalline polymers causing poor coalescence of the powder particles and hence fragile sintered parts [81]. This was also demonstrated in the work of Yang et al. [41], in which printlets sintered with amorphous polymers (PVA and HPMC) gradually softened, exhibiting a porous structure and a rough surface, whereas semi-crystalline polymers (PEG, stearic acid), which melt completely, showed a dense structure and a smooth surface. The authors also suggested that sintering was improved with polymers having a low melting/glass transition temperature and a wide stable sintering region (thermal gap between melting/glass transition point and degradation point). Furthermore, amorphous polymers have not been extensively studied, which explains the lack of data on their processing behavior [47]. They are not very numerous among the commercially available powders for SLS, although they constitute a large number of the pharmaceutical thermoplastic polymers.

From these differences in thermal properties, it can be concluded that sintering with pharmaceutical amorphous polymers would offer the advantage of producing dimensionally accurate printlets, improving process acceptability, but with poor mechanical properties. This was verified by printlets sintered with a semi-crystalline polymer “Kollicoat IR” which exceeded the limits of the hardness tester and could not break but only deform [27] while fragile printlets were printed when an amorphous polymer such as “Kollidon VA64” was used instead [29].

##### Extrinsic Properties

Critical extrinsic properties for printability include particle shape, granulometry, flowability and packing density [46]. It is important to highlight that none of these properties have been evaluated in the reviewed publications. Further studies will certainly need to be conducted to understand the impact of those properties on the sintering behavior.

The particle size of the powder used is critical to the resolution and the surface roughness of SLS manufactured parts, with larger particles being associated with higher surface roughness and low resolution [82]. Solid dosage forms printed with low resolution may deter patients to adhere to treatment. On the other hand, fine particles are often associated with high resolution but are not easily deposited on the building surface due to their poor flowability. In addition, they can be harmful for the safety of the printer as they can enter the internal parts of the machine and damage them [83]. Thus, if the pharmaceutical industries are considering manufacturing powders for SLS application, the powder particles will need to be in the optimal size range to utterly guarantee acceptable resolution, good flow and printer safety, i.e., 45–90 µm as suggested by Goodridge et al. [84].

#### 3.2.2. Active Pharmaceutical Ingredients (API)

Active ingredients were used mainly as tracers to assess the effect of SLS technology on the drug properties: thermal degradation, physical state and release. Although APIs are usually in an inferior proportion compared to the polymeric matrix, recently Yang et al. successfully sintered 2D structures containing only API without the use of a polymeric carrier [41]. Furthermore, printlets containing high-dose drug formulations [27,39,41] have shown that SLS is more tolerant of low polymer loading than FDM, where filaments prepared with 30% or less of polymer failed to be printable because they were too brittle [85].

Interesting to point out that sintering of APIs has been already explored with DDDs. These studies have helped to evidence the ability of some APIs such as Progesterone [54,58] and Ibuprofen [57] to absorb at the CO_2_ laser wavelength (10.6 µm) due to the presence of carbonyl groups and hence amplify the sintering.

#### 3.2.3. Fillers and Other Components

Besides thermoplastic polymers, actives ingredients and absorbance enhancers; more complex formulations integrating other components have been also explored. For example, lactose as a filler [33], mannitol as a taste masker [35], microcrystalline cellulose (MCC) as a filler-binder [42] and cyclodextrin complexes as a dissolution enhancer [35]. It is important to note that all the excipients mentioned above, did not interfere with the sintering process. Additionally, flow enhancers such as talc [39] and silicon dioxide [38] have been incorporated to overcome spreadability issues especially with drugs that may decrease powder flowability at high loading.

Although the use of multi-component blends can improve certain properties of the printlets, it can also be detrimental if there is low chemical affinity between the components. For example, immiscible blends of polymethyl methacrylate (PMMA) and polystyrene (PS) resulted in a decrease of the mechanical strength of printed DDDs [64]. Miscibility is therefore an important factor to consider when selecting the compounds that form the mixtures.

## 4. Variability of the Structure

### 4.1. Variability of the Macrostructure with the Design

**Figure 3:**
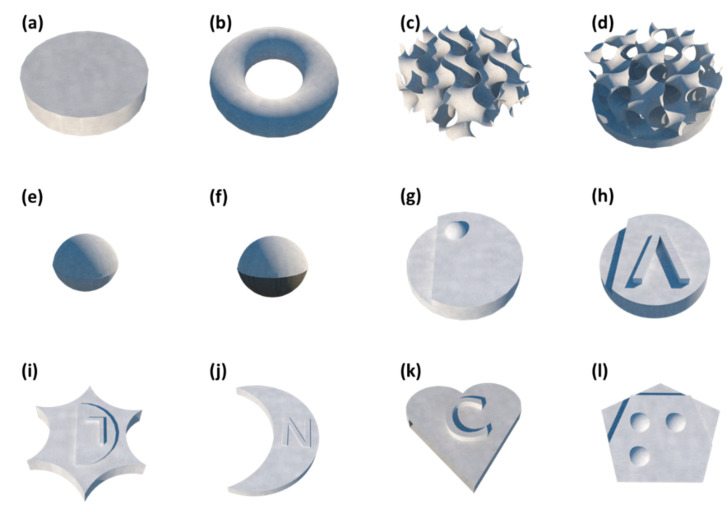
Different designs of dosage forms produced by SLS: (**a**) cylindrical [27,29,30,31,33,34,35,36,37,38,39,40,41], (**b**) torus [31], (**c**) gyroid lattice [30], (**d**) bilayer cylindrical and gyroid [30], (**e**) miniprintlet [32], (**f**) dual miniprintlet [32], (**g**) cylindrical with Braille A [36], (**h**) cylindrical with Moon A [36], (**i**) sun with Moon M [36], (**j**) moon with Moon N [36], (**k**) heart with Moon C [36], (**l**) pentagon with Braille M [36].

The ability to fabricate printlets presenting various geometries is not specific to the SLS technique but to 3D printing in general. However, precision varies from a printer to another and SLS shows good potential to produce highly complex structures with high resolution. The design of the reported SOFs varies from one study to another (Figure 3), depending on the intended application. For example, “Gyroid lattices” [30] which are complex porous structures generated by a special software, Flatt Pack^®^, offer a better way to tailor drug release for each individual by controlling the volume of voids within the structure prior printing. On the other hand, “miniprintlets” [32] are very small spherical dosage forms that do not exceed 2 mm in size, again confirming the finesse of the technique.

Changes in the macrostructure of the printlets had shown to affect their dissolution profile. For instance, compared to cylindrical printlets (Figure 3a), gyroid lattices (Figure 3c) showed faster drug release due to the important volume of voids [30]. The higher exposed surface area of gyroid lattices leads to rapid erosion of the polymer and hence faster dissolution of the drug. Moreover, bilayer printlets comprising a combination of gyroid lattice and cylindrical design (Figure 3d) exhibited an intermediate release rate. Therefore, this type of configuration offers a way to adapt drug release kinetics to the needs of individual patients by modifying the thickness and/or the number of the lattice layers. This approach was already explored with perforated dosage forms printed by FDM, *channeled tablets* [86] and *gaplets* [87]. As expected, faster drug release was achieved in the presence of wider channels. It should be noted that smaller gaps or channels may be obstructed using gelling polymers. This phenomenon can also occur with SLS printed dosage forms and should be taken into consideration when designing printlets.

Regarding the dissolution profile of miniprintlets (Figure 3e) [37], as expected, they liberated the API faster when the size was reduced. Even the water-insoluble ethyl cellulose matrices showed a drug release superior to 50% after 24 h. This was correlated with the high surface area of the spheres.

### 4.2. Variability of the Microstructure with the Process Parameters and the Material Attributes

#### 4.2.1. Porosity of Printlets Produced by SLS

It is also interesting to note that the internal structure is not only influenced by the pre-established design but also by the printing parameters. As in the case of FDM, where slicing parameters such as infill rate and infill shape could modify the geometry of printlets and hence influence their properties [88]; SLS printing parameters such as laser scanning speed can also affect the size, the form and the distribution of pores by modulating the degree of sintering [48].

For immediate-release dosage forms, porosity is critical and directly influences the disintegration process and the bioavailability of the API. In dosage forms where disintegration is absent, such as insoluble polymeric matrix systems, porosity remains a key factor for drug release as it conditions the penetration of the medium and the solvation of the API [89].

The impact of SLS on porosity has been the subject of numerous articles over recent years in non-pharmaceutical fields [90,91]. This parameter is principally studied to evaluate the mechanical properties [92] of printed parts which are critical for applications such as tissue engineering, automotive and aeronautics.

**Figure 4:**
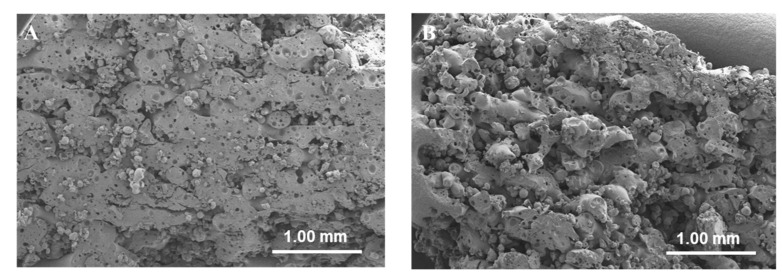
SEM images of vertical sections: (**A**) dense printlet, (**B**) porous printlet [78]. Reproduced with permission from the authors.

Porosity can be characterized by several different techniques such as helium pycnometry, mercury porosimetry, nuclear magnetic resonance (NMR), terahertz time-domain spectroscopy (THz-TDS) and X-ray microcomputed tomography (XµCT) [93]. However, in the reviewed articles, only XµCT was employed. It was used to visualize the internal structure of the printlets and to calculate closed and open porosity [27]. Open pores are connected to the external environment which allows direct diffusion of the medium and ensures a faster drug release [29]. On the other hand, closed pores are not in contact with the external environment and the dissolution medium cannot penetrate into them directly but it can diffuse through the polymeric matrix and form drug reservoirs [54]. Open pores are the voids between the poorly consolidated powder particles due to low sintering, whereas closed pores arise with excessive heating (high energy density) and are subsequent to gas pickups [94]. Scanning electron microscopy (SEM) provided visual confirmation of the porosity results obtained by XµCT. Typically, porous printlets show particles that can be easily distinguished however, denser printlets contain many molten areas (Figure 4).

#### 4.2.2. Critical Process Parameters and Material Attributes for Porosity in SLS

Studies on DDDs have largely explored the process parameters as well as the material properties influencing the porosity of the sintered parts. For instance, Leong et al. [95] attempted to produce DDDs with controlled-release profiles by modifying either the laser power or the scanning speed. The study demonstrated that high laser power or low scanning speed resulted in very narrow internal channels that ensured a more prolonged release of the drug. A linear relationship was found between porosity (assessed by mercury porosimetry) and laser power; however, this relationship was not consistent with scanning speed. Other studies have confirmed the effect of laser energy density on controlling the pore structure [56,63]. The bed temperature was also found to be determinant for the control of the porosity level and an inverse linear relationship was demonstrated between these two parameters [96].

Regarding critical material properties, it has been demonstrated that polymers with a high melt flow index (MFI) result in denser sintered structures. A study conducted on manufacturing blends of PMMA and PS by SLS; showed that by increasing the concentration of the component with the higher MFI (PS), porosity tended to decrease [64]. Moreover, the use of fine particles (106–150 µm) increased the superficial contact area, resulting in a higher degree of sintering and therefore less porosity [61,65,97].

In studies carried out with printlets, porosity was mostly composed of open pores [27]. These increased with the laser scanning speed due to the voids created between the unsintered particles, whereas the closed pores are more numerous at lower scanning speed when severe melting occurs but they remain negligible [29,39]. It was also found that increasing the loading of Paracetamol reduces the porosity [27] while increasing the percentage of Lopinavir increases the porosity despite using the same polymeric carrier “Kollicoat IR” [39]. This could be related to the fact that Paracetamol polymer melting whereas Lopinavir decreases it. On the other hand, increasing the filler loading (lactose or mannitol) [33,35] resulted in more porous printlets. This is mainly due to the high melting temperature of the fillers. Hence, filler particles remain in a solid state, maintaining the voids between them, and only the polymer is susceptible to melt and reduce the internal porosity.

### 4.3. Relationship between Porosity–Mechanical Properties and Drug Release

#### 4.3.1. Mechanical Properties

In general, hardness tended to decrease when the laser scanning speed was accelerated [29], or when the heating temperature was reduced [33]. Hence, the mechanical strength is directly related to the porosity, as porous printlets present weak interparticular bonding and break easily. This is consistent with previous results on the influence of high laser energy density on improving stiffness of the sintered DDDs [55,56,57,58,59,62,63]. It is, therefore, necessary to determine the optimal energy interval that balances between the mechanical integrity and stability of the drug [60].

The lowest hardness values (<15 N) were observed for orally disintegrating printlets (ODPs) [29,35,36,37]. As there are no minimum requirements for orally disintegrating tablets (ODTs) in terms of hardness, a necessary compromise between fast disintegration and tolerable aspect needs to be attained.

The incorporation of fillers or other components into the polymer matrix also showed an effect in the final mechanical properties by modulating the internal structure of the printlets. Although the presence of lactose decreased the mechanical strength of the printlets, MCC increased it due to its binding capability [33,37]. Previous studies with DDDs have also demonstrated this effect on the printed parts. For instance, Ibuprofen [57] had a hardening effect because it intensifies sintering, while Hydroxyapatite [97] and Fluorouracil [62] decreased the hardness of the DDDs because they space out the polymer particles hindering its continuous melting.

Furthermore, powder particle size has been found critical for the mechanical properties of DDDs [55,58,61,65]. Fine particles are more contiguous and as a result they form more junctions and decrease the porosity when they are submitted to the laser energy, which improves the mechanical strength of the printed parts. However, the effect of particle size on the degree of sintering has not yet been explored with printlets and will need to be taken into consideration in future work.

#### 4.3.2. Drug Release

**Figure 5:**
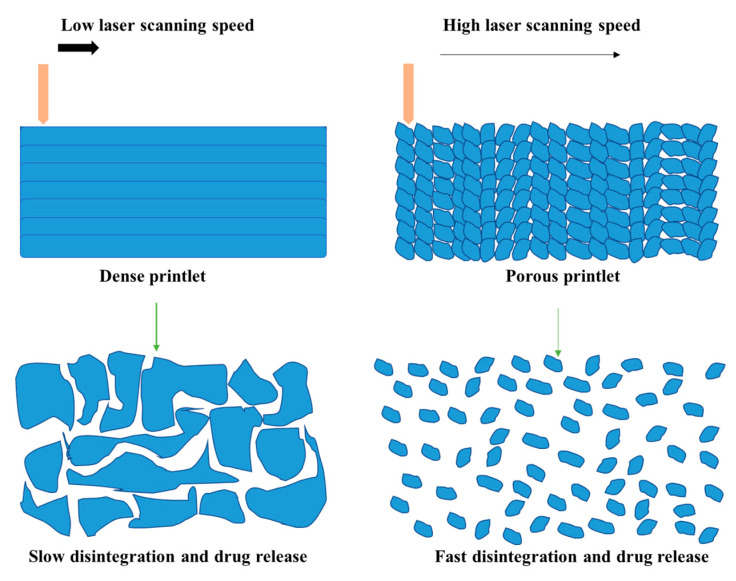
Relationship between laser scanning speed, porosity of the printlets and disintegration.

Dissolution profiles have shown that printlets released the API faster when open porosity values were higher, regardless of whether the drug release mechanism involved is erosion and/or diffusion [29,30]. In fact, open pores increase the accessible surface area allowing the medium to penetrate faster and deeper, which generally increases the rate of drug dissolution. Since increased filler loading [33,35,37] and accelerated laser scanning speed [29] are factors favoring the porosity, they consequently result in faster drug release. When printlets were prepared with an erodible polymer at high laser scanning speeds, they disintegrated rapidly into many fragments increasing the surface area and promoting fast dissolution of the API. For instance, the formulation prepared with Kollidon^®^ VA64 and Paracetamol printed at 100 mm/s took more than 600 s to disintegrate and reached complete drug release after 60 min [29]. Conversely, disintegration and drug release were achieved within 4 s and 10 min respectively, when the laser scanning speed was set at 300 mm/s. Figure 5 shows the relationship between laser scanning speed, porosity and disintegration for the case of erodible polymers such as Kollidon^®^ VA64.

In addition, it was found that temperature of the chamber is also a determinant factor for drug release, with higher temperatures implying more pronounced melting and lower pore density, and consequently slower drug release [33].

“Orally disintegrating printlets” (ODPs) [35] containing Ondansetron and mannitol exposed 90% of the drug release in 5 min and disintegrated in 15 s which is within the standards for ODTs according to the European Pharmacopeia (disintegration time inferior to 3 min) [98] and the FDA (disintegration time inferior to 30 s) [99]. This fast drug liberation is explained not only by their high porosity but also by the osmotic effect of the mannitol that allows a rapid imbibition of the dosage form. Moreover, porous ODPs with Braille and Moon letters were prepared and also showed a fast disintegration (~5 s) [36].

Porosity is the key asset of SLS for the pharmaceutical field because of its ability to produce fast disintegrating printlets unlike FDM or SLA [13]. The very fast disintegration times of ODP makes them very similar to the Spritam^®^ (11 s) [15], an ODT produced by an inkjet 3D printing technique, but with the important advantage that SLS is a solvent free process.

## 5. Amorphous Solid Dispersions (ASDs)

As previously mentioned, powders need to be continuously heated at the right temperature before and during sintering. The combination of these two thermal processes, heating and sintering, can enable the production of amorphous solid dispersions, [38,39] as has been demonstrated with other thermal production processes such as HME [100] or FDM [101]. This amorphization can enhance the dissolution and bioavailability of low solubility drugs (BCS Class II or IV) since the amorphous state is generally associated with higher solubility [102].

Difference scanning calorimetry (DSC) and X-Ray powder diffraction (XRPD) were used to determine the effect of sintering on the solid state of the material components by comparing the sintered printlets with their corresponding physical mixtures. DSC analysis showed that most of the printlets obtained by SLS presented a reduction or even a disappearance of the characteristic API melting peak. XRPD analysis generally corroborated the precedent results by demonstrating a more or less pronounced flattening of the specific crystalline peaks of the API. This suggests that the drug dissolves partially or totally into an amorphous form within the molten polymer during the sintering process [27,29,30,31,32,33,34,35,36,37]. These results highlight the potential of SLS for the fabrication of solid amorphous dispersions.

However, the degree of amorphization depends strongly on the nature of the polymer used. For instance, Eudragit L100-55 solubilized Paracetamol better than Kollicoat IR where it remained essentially crystalline. Likewise, a high percentage of crystalline drug could hinder amorphous conversion as demonstrated by Trenfield et al. [31]. However, the previously cited work did not have the premeditated aim of preparing ASDs, in contrast to the two studies recently conducted to specifically investigate the potential of SLS to produce ASDs, which will be discussed below.

The full amorphization of a poorly water-soluble drug, Ritonavir, with Kollidon VA64 using SLS was the first time described by Davis et al. [38]. The amorphization was confirmed by DSC, XPRD, Fourier transform infrared spectroscopy (FTIR) and using more sensitive techniques such as wide-angle X-ray scattering (WAXS) and solid-state NMR (ss-NMR). The FTIR analysis showed the appearance of hybrid peaks instead of overlapping peaks and the disappearance of NH and OH peaks, indicating weak molecular interactions and hence confirming the formation of the ASD. Solid-state NMR demonstrated broad peaks symbolic of amorphous forms and a small difference between the relaxation times of Ritonavir and Kollidon VA64, indicating a high degree of miscibility. Moreover, the printlets were comparable to the same formulations prepared by HME and even exhibited a higher degree of miscibility even though sintering is a zero-shear rate process. Finally, the ASD prepared by SLS showed a 21-fold concentration increase when compared to the physical mixtures, which confirmed the solubility enhancement of Ritonavir [38].

In a second stage, Hamed et al. [39] used chemometric models to quantify the crystallinity of Lopinavir is ASD prepared with Kollicoat IR by SLS. The chemometric models were based on the comparison of XRPD data of physical mixtures with identical composition to the printlets but presenting different crystalline/amorphous ratios. Multivariate analysis methods such as principal component analysis (PCA) and partial least squares (PLS) regression were used to validate the chemometric models. These models demonstrated a considerable amorphization of Lopinavir in the printlets ranging from 88.5% to 92.8%.

## 6. Applications of SLS in Personalized Medicine

3D printing is expected to transform personalized medicine due to its flexibility in producing printlets of different geometries and compositions. Currently, FDM is the most established additive manufacturing technique in pharmaceutical research, offering various ways to tailor the dosage and release of drugs to the individual needs of the patient. This is confirmed by the number of publications released in the last few years using this technology [28,103,104]. SLS is also holding the promise of personalized medicine through its findings about how process parameters and formulation properties affect porosity and drug dissolution [29].

**Figure 6:**
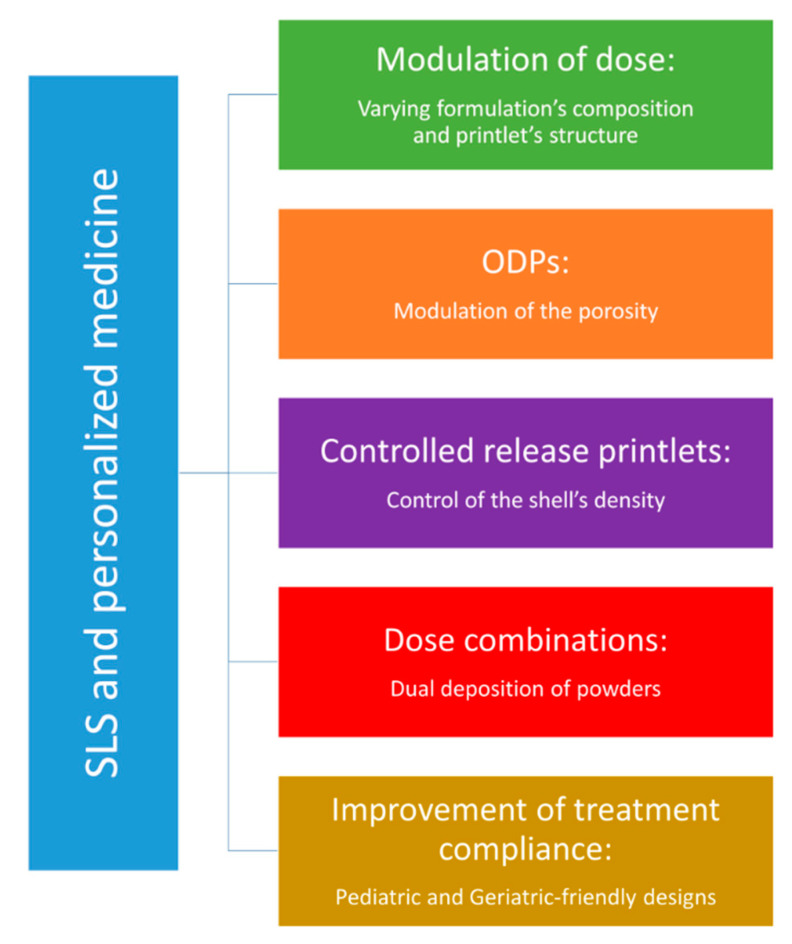
Applications of SOFs printed by SLS in personalized medicine.

Figure 6 summarizes the different applications of SOFs printed by SLS in personalized medicine, which will be discussed below.

### 6.1. Modulation of the Dose

In precision medicine, dosage control is as important as the release of the drug. For example, in FDM, dose tuning was achieved by varying either the drug ratio in the formulation, the size of the printlets or the internal porosity of the printlet (infill rate) [105,106]. However, in the case of SLS, only the modification of the formulation composition has been explored to control the dispensed drug dose [27]. This option implies that the printing feedstock will need to be changed from one patient to another requiring a different dosage of drug, thus generating more powder waste. Varying the dimensions of the printlet could be a good alternative, but high drug dosages will impose the production of large printlets that are difficult to swallow. Furthermore, printlet weight (and thus the drug dosage) has been shown to vary with the printing parameters laser scanning speed and heating temperature, correlated with the porosity modulation [33]. Although previous studies have demonstrated a linear relationship between infill rate and printlet weight in FDM [103,105], no evidence was found for a linear change in weight as a function of laser scanning speed in SLS [95]. Therefore, the main way to personalize the dosing in SLS with the printing parameters is to perform a pre-calibration step, which has not yet been reported.

### 6.2. Orally Disintegrating Printlets (ODPs)

As previously mentioned, porosity is the main advantage of this technology, as it allows fabrication of orally disintegrating printlets (ODPs) with individualized drug release profiles [29,35,36]. Regarding their mechanical properties, low hardness is a common characteristic of orally disintegrating forms and does not represent an issue with the proper conditioning. In addition, porosity is inherent to the energy density which controls the degree of melting of the particles. This parameter is also determinant for thermal degradation and amorphization of the API. For example, increasing the scanning speed would lead to a porous printlet with a short disintegration time, but at the same time it would only allow partial melting and thus a poor amorphization of the API. Disintegration and drug amorphization both contribute to improve dissolution of the API. For APIs with low solubility (BCS class II or IV), it would be interesting as future work to find the right compromise between these two characteristics to optimize API dissolution.

### 6.3. Controlled-Release Printlets

One way to control drug release in SLS is to fabricate printlets with a thick shell that could delay medium permeation and dissolution of the drug [107]. This technique has been used for DDDs featuring *dense wall formation* and *porous microstructures* and has demonstrated sustained release of a model dye [108]. The dense walls are generated by a common phenomenon in SLS known as *skywriting* [109], in which extra amount of energy is transmitted at the beginning and the end of a single line of sintered material due to the acceleration and deceleration of the laser beam. Therefore, composite DDDs are constituted of a dense outer shell and a porous matrix core. High laser power and slow scanning speed promote the formation of thick dense walls that delay dye release due to the low pore density and the poor pore interconnectivity. Further studies demonstrated that in order to build a sufficiently porous structure, the scanning line must be at least 2 mm long [96]. This could be transposed to the sintering of SOFs since many printers are now equipped with the option of scanning speed tuning for both the outskirt and the center of printed part allowing porosity stratification (a porous core and a thick shell). This pore size gradient could be achieved by applying an energy density gradient [97] but also with a particle size gradient [61]. If sustained release can be achieved, delayed release will require a placebo shell containing only polymer, which is difficult to implement in SLS compared to FDM where two different nozzles can be used. However, Yang et al. [41] printed sustained-release oral forms with an API-free shell and a drug-containing core, but no details were provided on how the two formulations were sintered at the same layer.

### 6.4. Dose Combinations

Dose combinations are also an interest of personalized medicine, as they contribute to minimize the number of medications taken per day and thus improve adherence to treatment and prescription compliance for fragile populations. The association of two or more drugs within the same polymeric matrix would require that all components withstand the applied temperatures and do not undergo thermal degradation. Conversely, the combination of two powders, each containing a different API associated with a polymer, would require that the two regions do not mix during printing. A concrete example of this approach is the *dual miniprintlets*, a dose combination of two drugs with different release properties depending on the polymeric carrier [32]. 

On top of fixed dose combinations, this approach could offer the possibility to develop *dynamic dose combinations*. These forms were explored by Sadia et al. in FDM [110] and demonstrated the possibility to titrate the dose of each API by controlling the number of deposited layers of each extruded filament prepared with a different formulation. However, as dual extrusion is not an available option on SLS, it could be replaced by *dual deposition*. This could be achieved automatically rather than manually (in the case of dual miniprintlets) [32], by having two reservoir platforms, each filled with a different powder feedstock. The printer would then execute the instructions dictated by the software. However, this would involve the ability of the printer to (i) switch from one powder reservoir to another, (ii) control the number of layers distributed of each powder, (iii) tune the printing parameters at each layer and (iv) pause between layers of different composition to permit the subsequent material to be heated to the right temperature. Such devices would help to raise the number of possibilities offered by SLS for pharmaceutical development.

### 6.5. Treatment Compliance

Finally, SLS could significantly improve treatment compliance within the fragile populations such as children, elderly, disabled patients, etc. This technique has already proven to be appropriate for the production of highly detailed structures such as printlets with Braille and Moon patterns. Researchers suggest that such forms could reduce medical errors and improve medication adherence for blind and visually impaired patients [36]. However, the identification of medicines by taking them out of their packaging instead of conventional identification from the Braille inscription on the primary or secondary packaging could pose other issues such as (i) contamination of the printlets by the user’s hands or the environment, (ii) degradation of the API due to moisture, (iii) breaking of the printlets due to their intrinsic low mechanical strength.

In the era of person-centered care, personalized medicine should not only take into account the physio-pathological profile of the patients but also their personal comfort. Palatability has been identified as the most compelling challenge for oral drug administration in children, while polypharmacy is the main barrier to compliance in the elderly [111]. SLS could resolve the issue of polypharmacy in geriatrics by providing dose combinations as mentioned above. In the case of children, Januskaite et al. [40] evaluated children’s visual preferences of SOFs printed by different techniques. Even though sintered printlets came second after those produced by digital light processing (DLP), they were described as more familiar, and children recalled their similar aspect to the medicines they usually take. In fact, SLS is unique as it produces printlets with a powdery surface reminding the aspect of tablets produced by conventional compression [44].

Nonetheless, while SLS is successful in printing a myriad of original geometric forms, it also has limitations. For example, its inability to produce chewable SOFs that are often preferred by children, as demonstrated by Januskaite et al. [40]. Another hurdle that can be associated with the process is the limitation of using colorants as they may interfere with the laser beam and preclude sintering or burn the printlets.

## 7. Technical Challenges

### 7.1. Printability of Pharmaceutical Materials

In SLS, the printability or ability of a powder to produce printed parts, needs to be considered before the desired characteristics of the printed parts. Both aspects depend on the properties of the powder in addition to the printing parameters. In other words, the fundamental study of raw materials and process parameters in SLS aims to define the critical properties for (1) manufacturing an object by laser sintering and (2) manufacturing a part by laser sintering with specific properties. The shift from one objective to another requires a change of perspective for certain polymer properties [47]. The critical material properties that can affect the quality of printed parts are more widely described in the literature than the properties necessary for successful sintering.

It is interesting to point out that among the 14 publications reviewed, only four of them detail the results of preliminary tests carried out to define the printing conditions [33,37,38,41]. The others just present the printing conditions that ensure optimal sintering without giving any further justification for the choice of settings. However, in order to understand the technology in detail and to allow a higher leap in pharmaceutical development, the potential printing constraints need to be exposed and explained; for example: the effect of colorant percentage on the degree of sintering (from low cohesion to excessive burning) [33,41]; solidification of the powder mixture at high chamber temperatures [33,38]; burning of printlets with high energy density [37,38,41] or percentage of fillers that improves flowability [37]. Moreover, no information on the printing yield has been reported. Batches of 10 to 100 printlets were programmed per print, but there was no indication on how many of these were successfully printed with minimal defects.

In general, publications on SLS of solid oral forms have mainly focused on evaluating the effect of printing parameters and material attributes on printlet properties; and only one publication [41] aimed to assess the printability of the raw materials, including drugs and excipients. This study demonstrated how both photo-absorber loading and energy density (ED) could be critical for the printing efficiency and dimensional accuracy. A high percentage of photo-absorber or an increase in the ED could certainly improve the printing depth but at the same time would lead to an important warpage of the printlet edges. In addition, ED must be set to ensure that sintered thickness is greater than the layer thickness, allowing the interconnection of subsequent printed layers. Furthermore, the study focused only on the intrinsic properties of the materials and did not take into consideration their physical properties. Therefore, in the future, further research on the printability of pharmaceutical materials is encouraged as it is important for the development of printable and generally recognized as safe (GRAS) materials.

### 7.2. Drug Stability

In general, high performance liquid chromatography (HPLC) has been used to verify that the drug content values were in agreement with the theoretical drug loading for the analyzed formulations and confirmed that no drug degradation occurred during the sintering process with a blue diode laser (445 nm) [27,29,30,31,32,33,34,35,36,37]. Other lasers such as CO_2_ lasers (10.6 µm), due to their high-power, could potentially degrade the drug or drastically modify the properties of the polymer. However, this argument can be contested as in many conventional SLS printers equipped with a CO_2_ laser, the power can be modulated, unlike in the Sintratec kit where this value is kept constant at 2.3 W. This was demonstrated by a more recent work published in 2021 (not reviewed) which reports successful CO_2_ laser sintering of printlets containing Copovidone and Paracetamol without degradation of the drug [78]. However, more sensitive active ingredients, such as proteinic hormones or enzymes, could be affected by the laser energy.

An interesting technique proposed to overcome denaturation of proteinic therapeutic agents is surface sintering. In this approach, only the shell of the dosage form is sintered, while the core remains unsintered. This technique was applied to a formulation of PLA, carbon microparticles and ribonuclease for scaffold sintering using a near infrared laser (λ = 0.97 µm) [60]. The polymer did not absorb but the carbon microparticles distributed on the surface did, protecting the enzyme located in the core from degradation and preserving its activity. Another alternative route is to load the drug into the oral dosage forms afterward by impregnation [95], which prevents the drug from interacting with the laser beam. However, drug impregnation may have limitations in terms of loaded quantities and homogenous distribution, as demonstrated for the filament impregnation in FDM [112].

### 7.3. The Need for Post-Processing

Printlets produced by SLS have a powdery aspect due the remaining unsintered particles of powder that did not fuse during sintering and stick to the surface [27]. This can cause a burst of drug release in some cases [27,30] as the unsintered particles of drug dissolve independently of the drug in the molten polymeric matrix. Furthermore, in another study, Raman spectroscopy was used to assess the solid-state uniformity of Paracetamol in a printlet produced by SLS [31]. The results revealed that in the core of the printlet, API was in the amorphous state whereas on the surface it was present in both amorphous and crystalline forms. The crystalline API corresponded to the residual unsintered powder particles of drug. These observations confirmed the need to properly brush-off the printlet after printing to ensure uniform distribution of the solid-state drug and to avoid dissolution irregularities.

### 7.4. Recycling

During the printing process, only part of the heated powder is sintered to build the intended object. To avoid waste, the unsintered powder is often reused for several printings. However, repeated heating is responsible for many issues including, thermal degradation, changes in the size and shape of particles due to agglomeration and increased molecular weight leading to an enhanced viscosity. Thus, the powder-recycling strategy can prevent optimal consolidation of the powder particles, which reduces the density and the mechanical performance of the printed part [48,81]. To overcome this problem, mixtures of recycled and virgin powders [92] and incorporation of stabilizing agents [81] are recommended to obtain a material with more stable thermal properties and to avoid varying the process parameters at each print. This cost-effective aspect is particularly important for pharmaceutical applications, especially when it comes to producing personalized printlets on demand which would only require a small portion of the powder bed. Therefore, the effect of powder recycling on the properties of the printlets would have to be determined in the future.

## 8. Regulatory Requirements for the Implementation of SLS

Despite the enormous progress made in the field of 3D printing of medicines, there are still regulatory questions to be addressed. In 2017, while guidelines for the additive manufacturing of medical devices [113] were issued by the FDA, no guidance for 3D printing of dosage forms was released. Similarly, no control assays specifically designed to assess the quality of 3D printed medicines are currently available in the different pharmacopeias. Thus, in the different studies investigating 3D printing technologies for pharmaceutical applications, classical control assays originally developed for products manufactured by conventional production processes were carried out.

New evaluation techniques need to be considered for 3D printed medicines, especially when the involved technologies use high temperatures. This thermal dimension, almost absent in conventional manufacturing processes, adds another line of control thermal degradation when it comes to 3D printing techniques. To control degradation or solid-state changes of the materials used in SLS, the incorporation of physicochemical characterization techniques such as thermogravimetric analysis (TGA); DSC and/or X-ray powder diffraction, for daily routine control, will need to be considered. The issue of the stability of the printlets over time should also be addressed, especially when oral forms are taken at a later stage. In this direction, more stability studies, such as that conducted by Hamed et al. [39], are encouraged as future investigations.

Quality by Design (QbD), a recent concept introduced by the Food and Drug Administration (FDA), could also be a forceful argument in favor of the implantation of 3D printing techniques into everyday clinical practice [114]. Design of experiments (DoE) is a QbD tool that helps to establish critical process parameters (CPPs) that affect critical quality attributes (CQAs). This approach has already been employed to determine the process and formulation variables that influence the properties of printlets produced by SLS [33,37].

Another QbD tool, Process Analytical Technology (PAT), could help to overcome the regulatory barrier of quality control. Control assays at the end of production are not rentable for on-demand manufacturing where only small quantities of dosage forms are produced specifically for a patient. This is where PAT is a good option by providing rapid, non-destructive analytical methods that allow an *at-line* control of the products and hence a real-time release (RTR) [115]. An example of qualitative PAT is Raman spectroscopy and mapping. This analysis has been used to evaluate both the physical state of the API and its distribution, within printlets printed in a SLS machine [31]. Near infrared (NIR) spectroscopy also falls under the umbrella of PAT as a portable NIR spectrometer enables the rapid dose verification. This approach is called point-and-shoot and was used to measure the concentration of API in printlets produced by SLS [31,34].

Nonetheless, some well-established assays could be adapted to new technologies, in particular to SLS, since it shares the same type of materials with direct compression and granulation. Indeed, Carr index and Hausner ratio calculated from bulk and tapped densities could be good values to estimate the flowability and compactness of powders and hence assess their suitability for the SLS process [46,78]. Eventually, the standards will need to be readjusted to meet the specific technological requirements. It is worth noting that unlike tableting, the powder’s low compactness would be more advantageous in SLS, especially for fast drug release forms where high porosity is desired. On the other hand, in a SLS printer, the powder is spread over the printing bed layer by layer rather than deposited in bulk, indicating that the optimal flowability may differ between techniques.

In fact, there is a wide variety of SLS printers available on the market which may differ in the type of laser beam, configuration of the reservoirs, printing resolution, type of slicer or software associated and in the number of controllable parameters. Therefore, along pharmaceutical grade powder feedstock, SLS printers will certainly need to be GMP certified or at least normalized to prevent reproducibility issues. It will have to be guaranteed that in any compounding pharmacy patients go to, they will receive the same personalized dosage form in terms of drug quantity, quality and bioavailability. For this to be possible, the technical differences between the future pharmaceutical SLS machines must be minimal or non-existent.

Moreover, the issue of microbiological quality could be addressed when it comes to drugs for human use, but the high processing temperatures typically used ensure that the printlets are generated in a germ-free environment. Likewise, filling the chamber with an inert gas (nitrogen) also contributes to the protection of printlets from contamination and hinders oxidative degradation [81,108]. Finally, SLS printers intended for pharmaceutical production will need to be manufactured in a way that facilitates cleaning, crucial step to avoid cross-contamination.

## 9. SLS between Pharmaceutical Industry and Compounding Pharmacies

The feasibility of sintering solid oral forms has been clearly demonstrated in the 14 publications reviewed, but what are the outcomes for the pharmaceutical field? Can the promises of this technology be fulfilled? How can we imagine the implementation of the SLS technique in pharmaceutical practice?

The remarkable ability of SLS to generate highly porous structures provides a very clear path of this emerging technology for the production of ODPs. Orally disintegrating forms show fast drug release, high bioavailability and good drug absorption. They are therefore primarily intended for patients who have difficulties swallowing solid dosage forms, such as pediatric and geriatric populations. Orally disintegrating forms can also be manufactured either by conventional pharmaceutical processes such as direct compression, freeze drying and spray drying, [116] or by more innovative technologies such as the patented 3D printing technology Zipdose^®^ used to manufacture Spritam^®^ [15]. Compared to those processes, SLS is a less complex manufacturing technique as it can be performed in a single step and it does not require the use of a solvent, which could be critical for some drugs. Nevertheless, SLS is not exempt of inconvenience, its excessive consumption of materials being the main drawback, especially when it comes to producing *one-off* batches in the precision medicine model. Hence, personalized medicine, which is meant to generate more savings by avoiding mass production of dosage forms, could not fulfill this guarantee and would instead lead to more waste of materials. On the other hand, mass production of printlets by SLS in the pharmaceutical industries would be more cost-effective but would not satisfy the paradigm of personalized medicine, *dose and drug release tuning*. So how can the pharmaceutical industry, compounding pharmacies, SLS technology and precision medicine articulate together?

An article published in 2019, proposed a model in which the pharmaceutical industry and the compounding pharmacies co-participate to implement FDM technology into routine clinical practice. Industries could mass-produce drug-loaded filaments that meet quality and safety requirements, while pharmacies, whether inside or outside hospitals, could transform formulated filaments into personalized printlets according to the patient prescription [69]. This could revolutionize the world of compounding by switching from classical tools *mortar and pestle* to digitalized engines *printers*. However, this schema does not fit SLS because unlike FDM there is no need to preprocess the printing materials. For this operation, powders could be directly purchased by compounding pharmacies and turned into printlets with a SLS printer. However, to overcome the high cost of excipients and APIs, pharmacies will have no choice but to recycle the powder. Thus, a careful control at any possible modification of the physicochemical properties of the already used material will need to be taken into account. This entails in particular: (i) accurate monitoring of the chamber temperature in order to detect any abnormal thermal fluctuation that could be harmful for the drug; (ii) control the particle size by sieving the powder before and after each printing to guarantee reproducible results; (iii) remixing the powder blend before printing to avoid any segregation between the different components; (iv) assessing molecular weight and viscosity variations of the polymers that could necessitate a reparameterization of the printing settings; (v) ensuring that drug/polymer ratio is kept constant or eventually correcting it; and finally and most importantly for the safety of users, (vi) controlling drug degradation. This last step could be realized either using traditional methods such as HPLC on samples regularly taken regularly from the powder feedstock, or by exploiting non-destructive analytical techniques that would avert exhaustion of the material.

**Figure 7:**
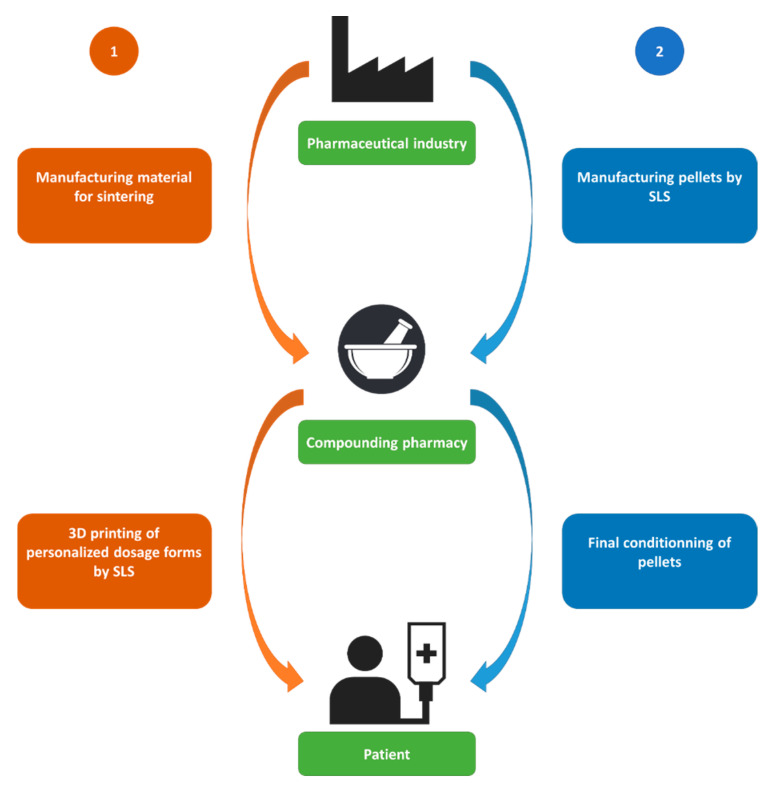
Collaboration of Pharmaceutical industries and compounding Pharmacies for the preparation of solid oral forms by SLS.

Nevertheless, SLS will not exclude the pharmaceutical industries out of the picture. First, just because sintering is feasible with powders already available on the pharmaceutical market does not mean that there is no need to improve them. Pharmaceutical industries could work on developing materials more suitable for SLS (Figure 7), which meet all the requirements in terms of laser absorbance, flow, compactness, particle shape and size distribution. They could eventually incorporate additives to improve flowability, enhance absorbance or protect polymers from oxidation during sintering. This reinforces the need to rethink pharmaceutical raw materials designated for new emerging process technologies and encourages future research to focus on understanding the sinterability of pharmaceutical polymers. Secondly, as suggested by Awad et al. [32] pellets or “miniprintlets” are more advantageous than single unit dosage forms on many levels. In fact, miniprintlets, such as other multiparticulate systems, generally present superior dosage flexibility and stability than oral liquid forms. In addition, by presenting a higher surface-to-volume ratio, they show better bioavailability, even in the presence of food in the gastrointestinal tract [117]. Furthermore, SLS exhibits better reproducibility than other methods of producing multipaticulate systems such as pelletization, granulation or spray drying. This is because the 3D printing technique can manufacture small pellets of regular size ensuring uniformity of the drug content and controlled-release properties. Thus, it seems quite reasonable to imagine that such systems could be produced on a large scale by the pharmaceutical industry and then conditioned in custom-made capsules or small bags by compounding pharmacies (Figure 7).

## 10. Conclusions

Selective laser sintering is a new chapter in 3D printing of solid oral forms and in individualized therapy in particular. Publications on the subject are not numerous, but they provide important insight on the technology and its benefits for personalized medicine. However, further studies are encouraged, especially in the field of printability of pharmaceutical polymers, which is a not well explored territory. In addition, there is a lack of information on how sintering might be affected by varying the laser wavelength, which was the same in all reviewed studies.

Porosity stands out as the main contribution of SLS technology, as both sintering parameters and material properties show the ability to modulate the internal structure of printlets. Therefore, orally disintegrating printlets appear as the most promising application for SLS of solid oral forms. Moreover, previous studies conducted on SLS of drug-delivery devices help to predict potential pharmaceutical applications such controlled-release printlets. In the long term, SLS could be an interesting asset for precision medicine, but in the meantime, there are still some technical and regulatory aspects to be addressed.

Finally, to prevent any quality and safety hazards for patients, guidelines such as GMP would need to be followed by pharmaceutical manufacturers in the future. QbD could accelerate the deployment of SLS in clinical practice by providing a better comprehension of the process and ensuring a real-time release test. Pharmaceutical industries, with their better knowledge of manufacturing processes, and compounding pharmacies, due to their proximity to end-users, could work hand in hand for concretizing personalized therapy by SLS.

## Figures and Tables

**Table 1:** Summary of the objectives, used materials, dimensions of the printlets and drug release profiles in the reported SLS studies for the production of SOFs.

Objective(s) of the Study	Polymers	Active Ingredients	Other Components	Dimensions of the Printlets	Drug Release Profile	References
To prove the suitability of SLS for the production of pharmaceutical oral dosage forms and its flexibility using different polymeric matrices and drug loadings (5%, 20%, 35%)	Kollicoat IR, Eudragit L100-55	Paracetamol		Cylindrical (10 mm diameter × 3.6 mm height)	Immediate and sustained	2017 [27]
Development of orally disintegrating printlets (ODPs) and optimization of the disintegration time by modulating the laser scanning speed	Kollidon VA64, Hydroxypropyl methylcellulose (HPMC)	Paracetamol		Cylindrical (10 mm diameter × 3.6 mm height)	Immediate and fast	2018 [29]
Development of complex porous structures “gyroid lattices” offering a tailored drug release	Polyethylene oxide (PEO), Eudragit RL, Eudragit L100-55, Ethylcellulose	Paracetamol		Cylindrical and gyroid lattice (10 mm diameter × 3.6 mm height)	Fast, immediate and sustained	2018 [30]
Applicability of PAT techniques (NIR spectroscopy and Raman mapping) on sintered dosage forms to facilitate the integration of 3D printing in clinical practice	Eudragit L100-55, HPMC	Paracetamol		Cylindrical (10 mm diameter × 3.6 mm height), torus (10 mm diameter × 4 mm height) and square (10 mm side and 0.5 mm thickness)	N.A.	2018 [31]
Demonstration of SLS high resolution by printing small oral dosage forms “miniprintlets” and dose combinations of two drugs with different release properties	Kollicoat IR, Ethylcellulose	Paracetamol, Ibuprofen		Spherical (1- and 2-mm diameter)	Immediate and sustained	2019 [32]
Determination of the effects of process parameters and filler loading on the quality of printlets using a DoE	Kollidon VA64	Diclofenac sodium	Lactose monohydrate	Cylindrical (10 mm diameter × 3 mm height)	Immediate	2019 [33]
Suitability of NIR spectrometry for the simultaneous dosage verification of two drugs within polyprintlets	PEO	Amlodipine, Linisopril		Cylindrical (10 mm diameter × 3.6 mm height) and square (10 mm side and 0.5 mm thickness)	N.A.	2020 [34]
Fabrication of personalized ODPs by incorporating drug-cyclodextrin complexes combined with mannitol	Kollidon VA64	Odansetron	Mannitol, cyclodextrin	Cylindrical (12.4 mm diameter × 3.6 mm height)	Fast	2020 [35]
Development of ODPs with Braille and Moon patterns to improve identification for blind and visually impaired patients	Kollidon VA64	Paracetamol		Cylindrical (10 mm diameter × 3.6 mm height)	Fast	2020 [36]
Sintering optimization by studying the effect of laser scanning speed and concentration of excipients	Kollidon VA64	Clindamycin palmitate hydrochloride	Lactose monohydrate and microcrystalline cellulose	Cylindrical (10 mm diameter × 3 mm height)	Immediate	2020 [37]
Improvement of poorly water-soluble drug solubility by developing amorphous solid dispersions with SLS	Kollidon VA64	Ritonavir	Silicon dioxide	Cylindrical (10 mm diameter × 4 mm height and 12 mm diameter × 5 mm height)	Sustained	2020 [38]
Evaluating the effect of formulation and SLS printing parameters on drug crystallinity using chemometric models	Kollicoat IR	Lopinavir	Lactose monohydrate, talc	Cylindrical (4.5 mm diameter × 3 mm height)	Fast and immediate	2020 [39]
Investigation of the visual preferences of printlets produced by four different 3D printing techniques (including SLS) among the pediatric population	Kollicoat IR			Cylindrical (10 mm diameter × 3.6 mm height)	N.A.	2020 [40]
Evaluation of the printability of different drugs and polymers by SLS with various absorbance enhancers and printing parameters	Eudragit EPO, Polyvinylalcohol (PVA), Polyethylen-glycol (PEG), Carboxymethyl starch sodium, Eudragit RL, Stearic acid, HPMC, Ethylcellulose, Kollicoat MAE	Indomethacin, Nifedipine, Astragalus polysaccharin, Ibuprofen, Metoprolol, Tinidazole, Paracetamol, Diclofenac sodium, Bletilla striata		2D structures (0.5 mm height): circle (10 mm diameter), triangle (12 mm side), honeycomb (10 mm diameter), moon (10 mm length), star (14 mm length), number 1 (10 mm length); and their respective 3D printlets (4 mm height)	Sustained and immediate	2020 [41]

N.A.: non-applicable.

## Data Availability

Not applicable.
